# Case Report: A very rare case of diffuse large B-cell lymphoma with cardiac and ovarian involvement

**DOI:** 10.3389/fonc.2025.1531668

**Published:** 2025-06-11

**Authors:** Yadan Du, Yuting Tian, Yawen Chen, Shuaihua Cheng, Jianping Gao

**Affiliations:** ^1^ Department of Surgical Oncology, Gansu Provincial Hospital, Lanzhou, China; ^2^ The First School of Clinical Medicine, Gansu University of Chinese Medicine, Lanzhou, China

**Keywords:** diffuse large B-cell lymphoma, non-Hodgkin lymphoma, heart, ovary, case report

## Abstract

**Background:**

Diffuse large B-cell lymphoma (DLBCL) is the most prevalent type of aggressive lymphoma, commonly spreading to sites such as the lymph nodes, spleen, bone marrow, liver, lungs, and central nervous system. However, metastasis to the heart and ovaries is relatively uncommon.

**Case Description:**

A 63-year-old woman visited the hospital with abdominal pain and bloating, but showed none of the typical signs of lymphoma. Imaging scans revealed abnormal masses in both the pericardium and ovaries. A biopsy confirmed it was DLBCL, presenting in the rare form of simultaneous spread to the heart lining and ovaries. During the course of illness, she also developed atrial arrhythmia. Doctors adopted a phased treatment approach: four cycles of R-CEOD chemotherapy led to a noticeable reduction in the heart tumor and improvement in her heart rhythm. This was followed by four cycles of R-CHOP, which further shrank the cardiac lesion and cleared the abdominal tumors completely. The treatment was well tolerated, and at a three-month follow-up, there was no sign of recurrence. Her heart function remained stable, with a left ventricular ejection fraction (LVEF) of 60%.

**Conclusion:**

This case highlights the importance of early detection of atypical metastases in DLBCL through a combination of various imaging and pathological tests. Additionally, personalized treatment strategies may contribute to better patient outcomes.

## Introduction

Diffuse large B-cell lymphoma (DLBCL) is the most prevalent form of aggressive non-Hodgkin lymphoma (NHL), representing 30-40% of all NHL cases (1, 2). DLBCL arises from B cells and is marked by the uncontrolled growth of tumors resulting from the monoclonal proliferation of abnormal lymphocytes (1). This type of lymphoma often metastasizes to the bone marrow, spleen, liver, bones, and central nervous system (3, 4). While DLBCL can occur throughout the body, metastasis to atypical sites like the heart and ovaries is exceedingly rare (5).

Cardiac metastasis is an uncommon complication of DLBCL, typically affecting the right atrium, right ventricle, pericardium, or major blood vessels of the heart (6). Patients may experience symptoms such as chest pain, difficulty breathing, or fainting (7). In severe cases, this can result in cardiogenic shock or arrhythmias. Because these symptoms are often subtle, early diagnosis of cardiac metastasis can be quite difficult, and the prognosis is usually poor. Imaging techniques, like PET-CT or cardiac ultrasound, are essential for diagnosis (8, 9). Although chemotherapy can help reduce tumor size to some extent, the overall mortality rate remains high (10). The ovaries are another uncommon site for DLBCL metastasis, potentially resulting from abnormalities in the local immune environment or through spread via the bloodstream (11). Ovarian metastasis usually manifests as nonspecific symptoms like abdominal bloating or lower abdominal pain, making it easy to confuse with other ovarian issues and complicating the diagnosis (12).

This article presents a rare case of DLBCL with multiple site metastases, where the lymphoma spread to both the retroperitoneal lymph nodes and affected the heart and ovaries. By thoroughly analyzing the clinical presentation, imaging characteristics, diagnosis, and treatment of this case, we hope to offer valuable insights for clinicians dealing with rare instances of multiple site metastasis in DLBCL. This could improve their ability to identify and manage such complex cases.

## Case presentation

### Chief complaint

The patient has been experiencing persistent abdominal pain and bloating for the past two weeks, along with a stoppage of bowel movements and gas for the last two days.

### Present illness

The patient developed abdominal pain and bloating without any obvious cause about two weeks ago, mainly focused in the upper abdomen, described as a constant dull ache. The symptoms are accompanied by a loss of appetite, fatigue, occasional acid reflux, and heartburn, as well as sensations of cold and chills, although the patient’s temperature is normal. The patient exhibits mild facial swelling but reports no other significant discomfort. Five days ago, the abdominal bloating intensified and spread to the entire abdomen. Two days ago, the patient stopped having bowel movements and passing gas, and also started experiencing shortness of breath and difficulty breathing. Since the onset of the condition, the patient has not noticed any significant weight changes.

### Past medical history

The patient has no known chronic illnesses, no history of surgery or use of special medications, and does not smoke or drink alcohol. There is no family history of cancer or related diseases.

### Physical examination

The skin and sclera appear normal, with no signs of jaundice or pallor, and there is no palpable enlargement of the superficial lymph nodes. Breath sounds in both lungs are clear, with no dry or wet rales. The heart rhythm is regular, and there are no abnormal murmurs detected in any of the valve auscultation areas. The abdomen is flat and exhibits tenderness across the entire area, with no rebound tenderness or muscle rigidity. The liver and spleen are not palpable below the ribs, and the kidneys are also not felt. Liver dullness is noted, but there is no shifting dullness. Both kidney areas show no tenderness on percussion. Bowel sounds are reduced, and no gurgling sounds are heard. There is no swelling in either lower leg.

### Laboratory findings

Complete Blood Count: The patient’s blood test showed a higher-than-normal white blood cell count (12.5 × 10^9/L), mainly due to a significant rise in neutrophils (9.04 × 10^9/L). Monocytes were also slightly elevated (1.22 × 10^9/L). Although the percentage of lymphocytes was lower than usual (17.7%), their absolute number remained within the normal range (2.21 × 10^9/L), suggesting no true deficiency. Eosinophils were reduced (0.2%), with their count nearing the lower normal limit (0.02 × 10^9/L). No abnormalities were found in other cell types, such as basophils.

Biochemical Results: Lactate dehydrogenase is 782.79 U/L, NT-proBNP is 2052.00 pg/ml, and high-sensitivity cardiac troponin I (hsTnI) is 0.0540 ng/ml. Interleukin-6 is 132.67 pg/ml, and procalcitonin is 0.390 ng/ml.

### Imaging studies

**Figure 1:**
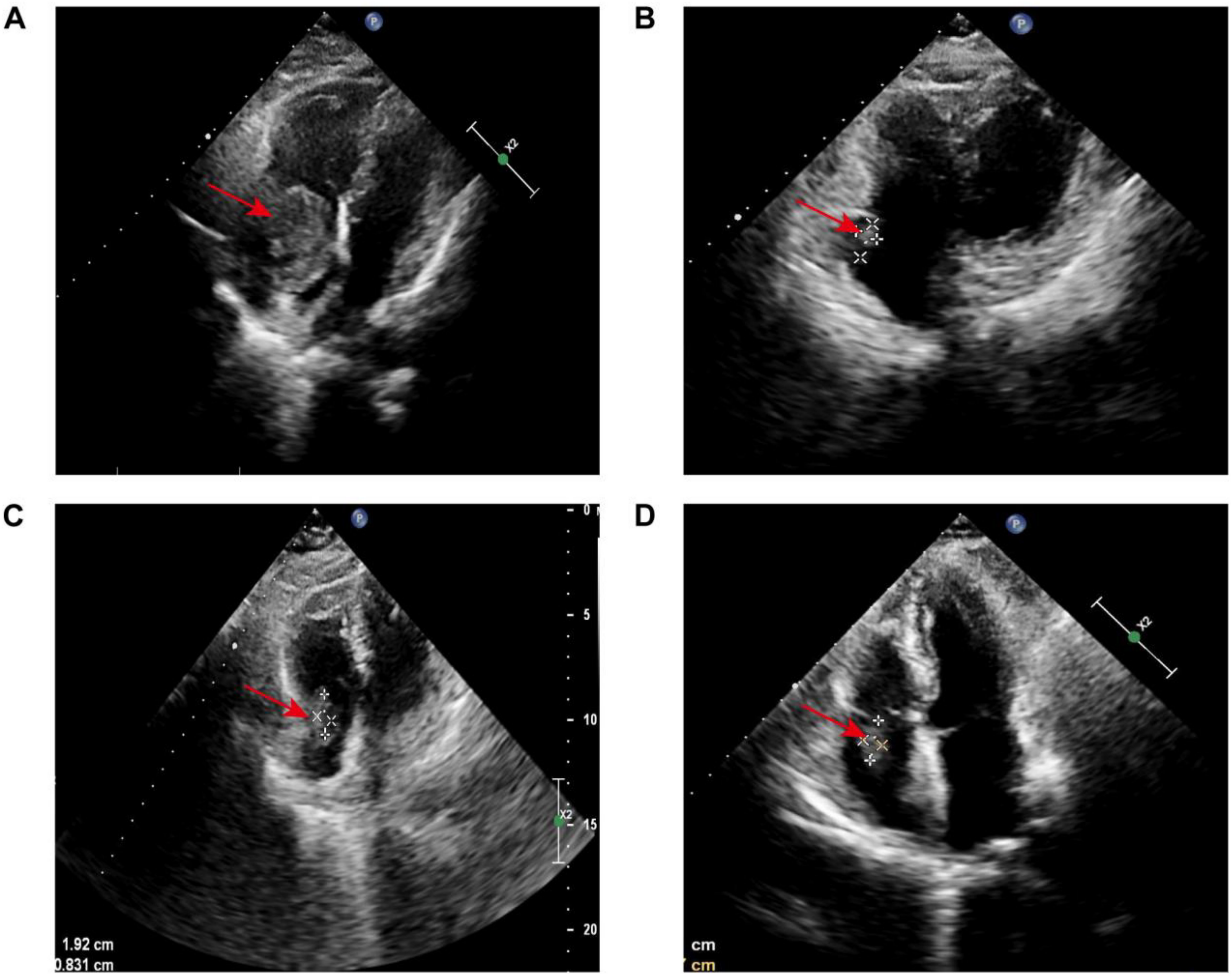
Serial echocardiographic surveillance of cardiac lesion: **(A)** Initial transthoracic echocardiography revealed a roughly oval-shaped filling defect within the right atrium, measuring approximately 43 × 34 mm. The lesion appeared to be anchored to the superior aspect of the right atrial wall. It demonstrated well-defined margins and minimal mobility, suggestive of a space-occupying mass with low dynamic activity. **(B)** Following four cycles of R-CEOD chemotherapy, reevaluation revealed a 14 × 8 mm hypoechoic-to-isoechoic lesion along the posterolateral wall of the right atrium. The lesion remained well-circumscribed, with a near-oval contour and persistently low mobility. **(C)** After an additional four cycles of R-CHOP, imaging showed the lesion to be stable in size at 19 × 8 mm. Its echogenicity remained mixed hypoechoic, and morphological characteristics (including its oval shape, sharp margins, and low mobility) were consistent with previous findings. The lesion continued to localize to the posterolateral wall of the right atrium. **(D)** At a three-month post-chemotherapy follow-up, the lesion measured 18 × 7 mm. Its acoustic properties and morphological features (mixed hypoechoic pattern, oval shape, well-defined borders, and limited mobility) remained unchanged. The basal attachment site was concordant with prior assessments.

Cardiac ultrasound: The effective volume of the right atrium is decreased, showing a circular filling defect about 43 × 34 mm in size, likely attached to the top of the right atrium, with clear borders and minimal movement (**Figure 1A**). Additionally, a low-echo area measuring approximately 18 × 12 mm is noted near the right atrioventricular junction.

**Figure 2:**
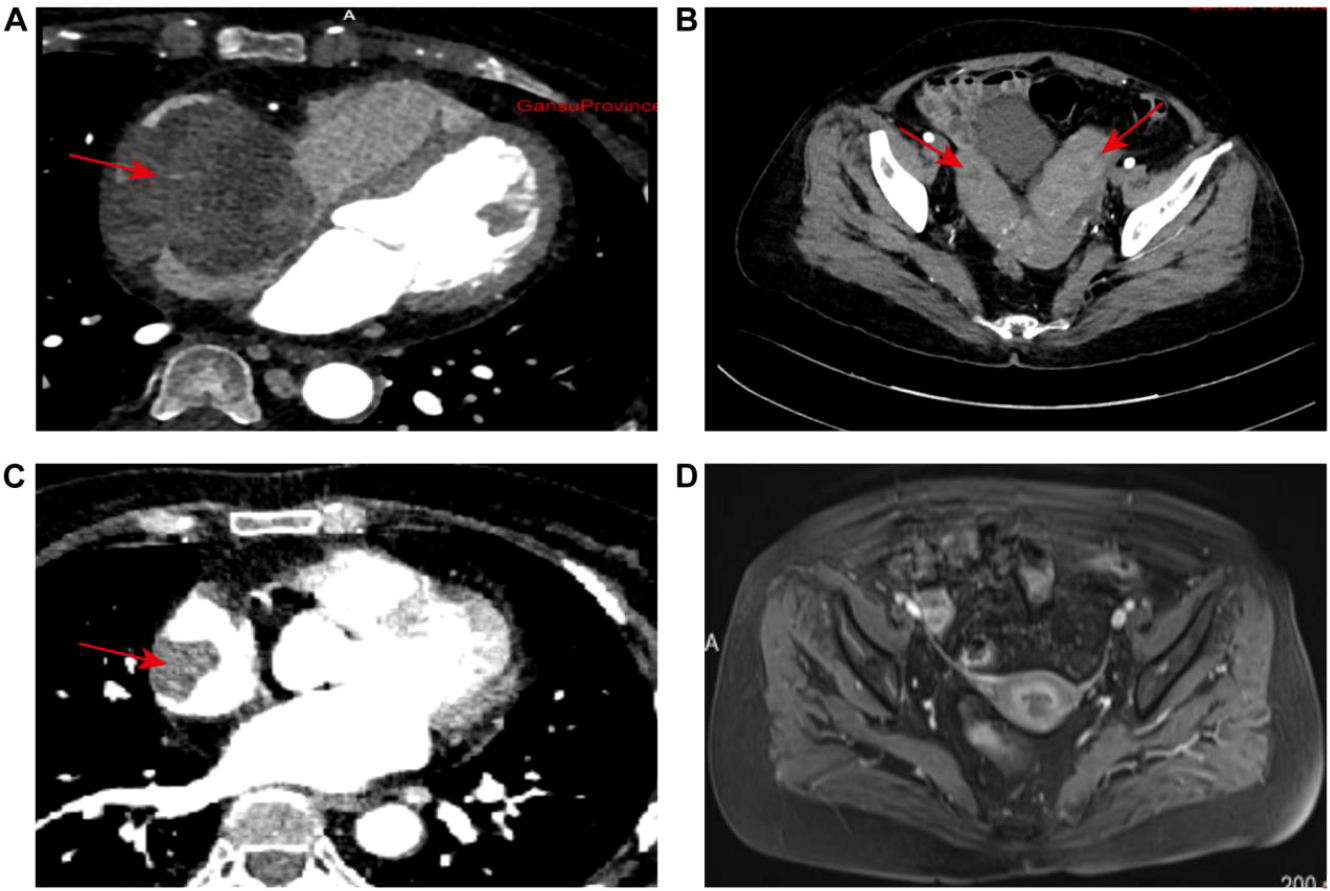
Radiological evaluation of disease dynamics: **(A)** Baseline imaging revealed marked enlargement of the right atrial chamber. A lobulated, mixed-density mass measuring approximately 6.5 × 5.6 cm was identified at the junction of the right atrium and pericardium. The lesion exhibited well-demarcated borders and demonstrated pronounced heterogeneous enhancement on contrast-enhanced scans. **(B)** Initial pelvic imaging identified bilateral adnexal masses characterized by well-defined mixed densities. The largest lesion measured 42.4 × 73.5 × 50.0 mm. Dynamic contrast-enhanced imaging revealed a pattern of persistent enhancement, interspersed with multiple non-enhancing patchy areas throughout the lesion. **(C)** Post-standard chemotherapy re-evaluation showed significant regression of the primary right atrial lesion, with a maximal axial dimension of 30 × 25 mm. Imaging characteristics were consistent with a localized filling defect. **(D)** At the final post-chemotherapy pelvic follow-up, complete resolution of bilateral adnexal lesions was observed. No abnormal enhancement or space-occupying lesions were detected. The imaging data comprehensively captured the temporal evolution of lesion morphology and enhancement characteristics throughout the treatment course.

Enhanced CT: The right atrium is markedly enlarged, with a lobulated, well-defined mass of mixed density seen in the right pericardium and right atrium, measuring about 6.5 × 5.6 cm (**Figure 2A**). The contrast scan shows uneven enhancement. The mass extends from the right atrial appendage down to the entrance of the inferior vena cava. Near the phrenic angle, two enlarged lymph nodes are visible, with the larger measuring approximately 2.7 cm and showing significant enhancement with contrast. There are multiple mildly enlarged lymph nodes in both the pulmonary hila and the mediastinum. Additionally, several enlarged lymph nodes are seen near the major blood vessels in the retroperitoneum, with the largest measuring about 2.1 cm. In the adnexal region, a mixed-density mass is observed, with the larger one measuring about 42.4 × 73.5 × 50.0 mm (**Figure 2B**). The enhanced scan shows significant and sustained enhancement, with scattered patchy areas within the lesion that do not enhance.

### Pathology findings

**Figure 3:**
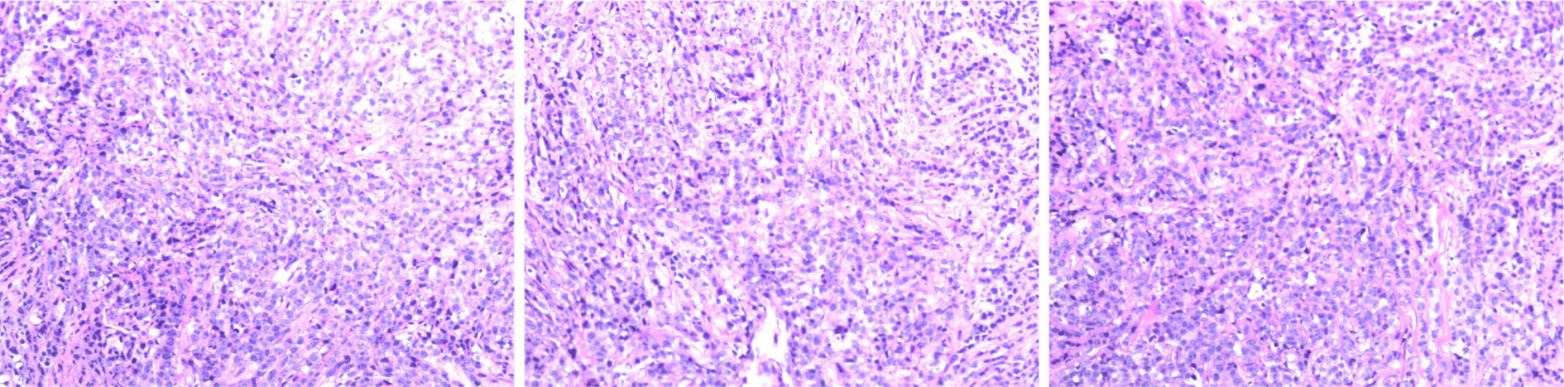
Pathological slide: Diffuse large B-Cell Lymphoma (DLBCL, NOS, Non-GCB subtype).

Immunohistochemistry: Positive for CD20, CD79, BCL-2 (60%), BCL-6, MUM1, and C-myc (40%); negative for Cyclin D1, with a Ki67 proliferation index of 70%. These results are consistent with the characteristics of non-Hodgkin DLBCL (**Figure 3**).

**Figure 4:**
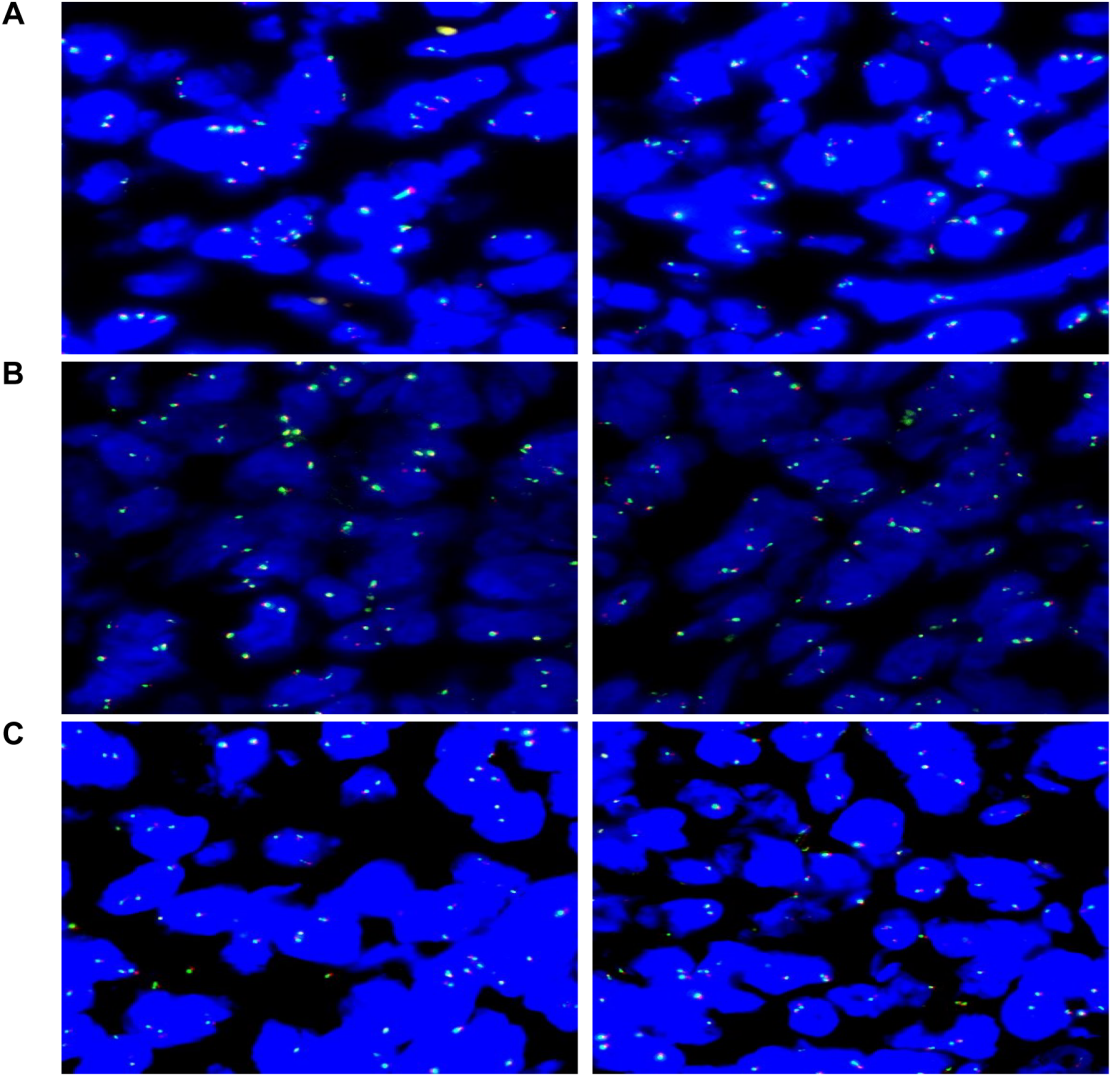
Molecular pathology diagnostic report: **(A)** Analysis of 100 tumor cells shows that 7% exhibit abnormal signal patterns, remaining below the 15% threshold, indicating no MYC gene rearrangement (negative). **(B)** Analysis of 100 tumor cells shows that 4% exhibit abnormal signal patterns, remaining below the 15% threshold, indicating no BCL6 gene rearrangement (negative). **(C)** Analysis of 100 tumor cells shows that 3% exhibit abnormal signal patterns, remaining below the 15% threshold, indicating no BCL2 gene rearrangement (negative).

Genetic Testing: FISH analysis reveals no breakages in BCL2, BCL6, or MYC (negative) (**Figures 4A–C**). Clonal gene rearrangement testing suggests a monoclonal proliferation of B cells.

### Final diagnosis

Non-Hodgkin lymphoma, B-cell type, stage IV, with involvement of the retroperitoneal lymph nodes, heart, and ovaries.

### Treatment plan

**Figure 5:**
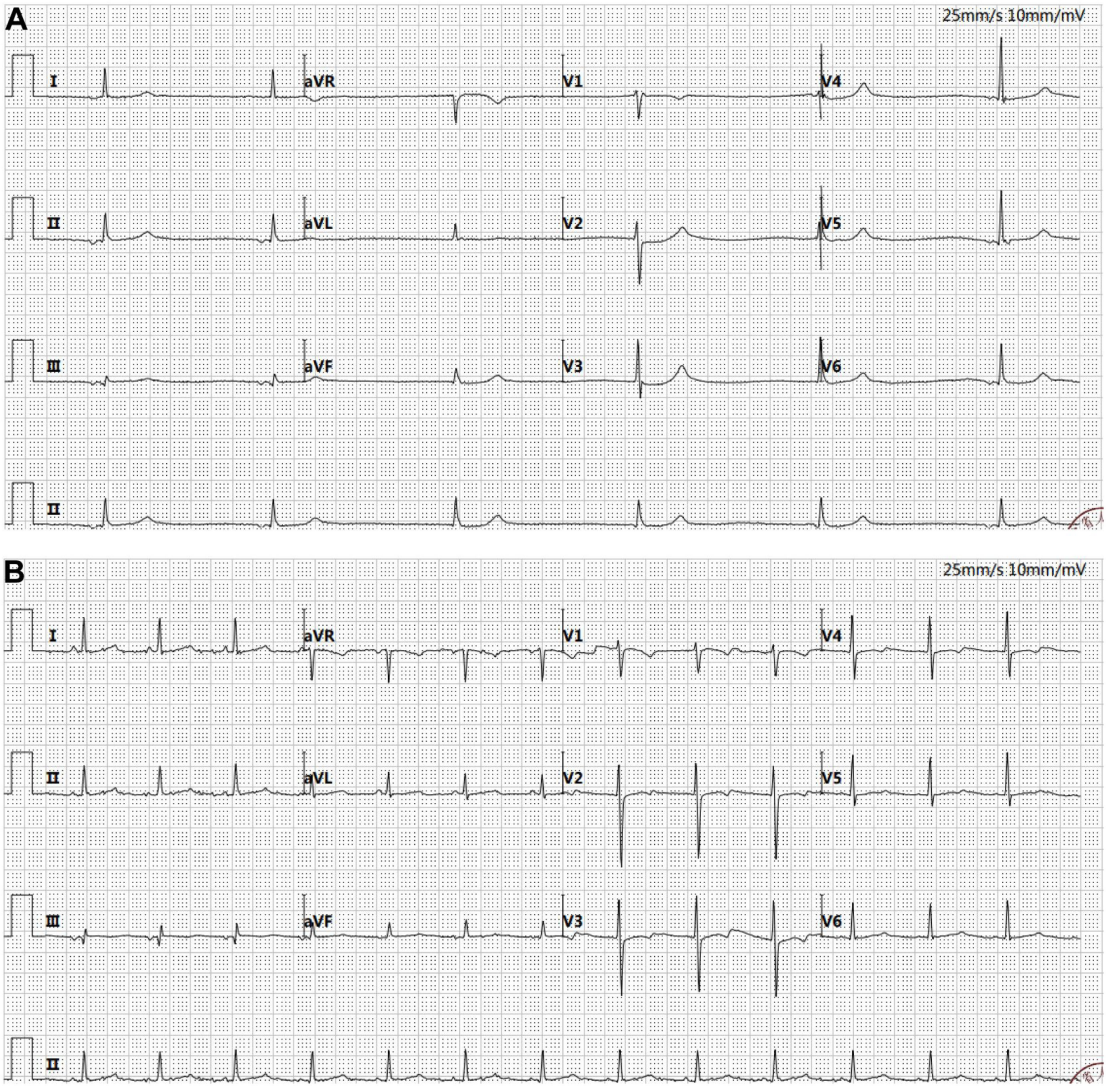
Electrocardiogram. **(A)** Atrial escape rhythm (36 bpm) and junctional escape rhythm were observed. The QT interval was prolonged (525 ms). ST segment depression of 0.05–0.1 mV was noted in leads V2–V4. RV5 amplitude was elevated at 1.28 mV, with a combined RV5 + SV1 value of 1.88 mV. The P wave duration was 82 ms, and the QRS complex measured 94 ms. **(B)** Sinus rhythm was restored (80 bpm). The QT interval normalized to 410 ms, and the ST segment changes resolved. RV5 amplitude decreased to 0.9 mV, with a combined RV5 + SV1 of 1.57 mV. The P wave measured 74 ms, and the QRS complex was 84 ms.

According to the guidelines, the patient met the criteria for the R-CHOP chemotherapy regimen. However, during treatment, the patient developed arrhythmias (with ECG showing junctional arrhythmia) (**Figure 5A**) and exhibited an increased risk to heart function. Given these cardiovascular concerns, the decision was made to switch from the R-CHOP regimen to the R-CEOD regimen to reduce cardiac toxicity and ensure the patient’s safety. This regimen consisted of rituximab 600 mg, cyclophosphamide 1200 mg/m², pirarubicin 60 mg/m², and etoposide 100 mg/m², administered on days 1 through 3. Following four cycles of standardized antitumor therapy, the right atrial mass exhibited a marked reduction in size, with no recurrence of arrhythmias or other cardiac adverse events. Cardiac function parameters remained stable. Follow-up transthoracic echocardiography revealed an intracavitary right atrial mass measuring 14 × 8 mm, presenting as a heterogenous hypoechoic to isoechoic lesion with an irregular three-dimensional morphology, well-demarcated margins, and significantly restricted mobility (**Figure 1B**). Serial electrocardiographic monitoring demonstrated persistent sinus rhythm, and no evidence of atrial tachycardia, atrial fibrillation, or other abnormal atrial electrical activity was detected on ambulatory Holter recordings (**Figure 5B**). Following a reassessment during follow-up, the treatment was transitioned to the guideline-recommended R-CHOP regimen (cyclophosphamide, doxorubicin, vincristine and prednisone) to further enhance efficacy. Following four cycles of treatment, imaging assessment revealed a marked reduction in tumor burden. Transthoracic echocardiography showed a substantial decrease in the size of the right atrial mass, with a maximal diameter of 19 × 8 mm on two-dimensional imaging (**Figure 1C**). Color Doppler flow imaging did not reveal any evidence of inflow tract obstruction, the left ventricular ejection fraction (LVEF) is 62%. In contrast, contrast-enhanced CT demonstrated a larger filling defect in the right atrium, measuring up to 30 × 25 mm in maximal cross-sectional area (**Figure 2C**), highlighting discrepancies between anatomical measurement modalities. According to RECIST 1.1 criteria, the sum of diameters of target lesions indicated a partial response. Abdominal re-imaging showed complete resolution of the previously noted peritoneal metastases (**Figure 2D**), indicative of a heterogeneous response across disease sites. Given the persistence of the intracardiac mass and limitations of conventional imaging in accurately assessing residual disease, a multidisciplinary team recommended PET-CT to evaluate metabolic activity and differentiate between viable tumor and treatment-related changes. However, the patient declined this investigation.

### Planned follow-up

According to the standardized post-treatment surveillance protocol for tumors, the patient requires regular multimodal imaging assessments every three months, alongside evaluations of cardiac function. Key monitoring indicators include dynamic changes in LVEF, aimed at the early detection of chemotherapy-induced cardiotoxicity and signs of tumor recurrence. At three months following the final chemotherapy cycle, imaging review revealed a right atrial mass on echocardiography measuring 18 × 7 mm, characterized by hypoechoic to isoechoic signals, well-defined margins, and limited mobility (**Figure 1D**). LVEF remained preserved at 60%, suggesting compensated cardiac function. Longitudinal comparison indicated that fluctuations in the maximal diameter of the mass were within the range of technical variability. According to RECIST 1.1 criteria, given that the sum of the maximal diameters of target lesions had not increased by 20%, the current therapeutic response is assessed as stable disease.

## Discussion

This case illustrates the rare metastasis of DLBCL to the heart and ovaries, underscoring the highly aggressive nature and unusual metastatic patterns of this lymphoma. The uniqueness of this case lies in the simultaneous involvement of both the heart and ovaries, a combination rarely reported, offering valuable insights into the metastatic patterns of DLBCL. While DLBCL commonly metastasizes to the bone marrow, liver, spleen, and central nervous system, its spread to the heart and ovaries is unusual (3, 11, 13). In clinical practice, metastatic lesions in the heart and ovaries are often subtle and usually lack early symptoms, making early diagnosis particularly challenging (7, 14–16).

While there are many reports on the metastatic patterns of DLBCL, the specific mechanisms underlying its spread to the heart and ovaries remain insufficiently studied (17, 18). Cardiac metastasis is typically thought to reach the pericardial cavity via the bloodstream or direct infiltration (19), while ovarian metastasis may be linked to hematogenous or lymphatic spread (20). Furthermore, more aggressive subtypes of DLBCL may develop metastatic lesions in tissues distant from the primary site through mechanisms like invasion of vascular endothelial cells or immune evasion (21–23). In this case, the imaging findings of masses in the right atrium and right pericardium suggest that DLBCL may preferentially target areas with abundant blood supply, particularly on the right side of the heart.

The imaging features in this case clearly revealed distinct lesions in the heart and ovaries, offering critical imaging evidence for diagnosing multi-site metastasis in DLBCL. The cardiac metastasis presented as a mixed-density, lobulated mass with clear borders in the right atrium, while the ovarian metastasis appeared as an unevenly dense mass in the adnexal regions. Based on previous reports of DLBCL imaging findings, we speculate that there may be a pattern in the imaging characteristics of multi-site metastasis (24–26). To enhance the sensitivity of imaging in diagnosing multi-site metastasis in DLBCL, future efforts could focus on creating a standardized system for evaluating DLBCL imaging features, particularly with standardized descriptions for metastasis to rare sites like the heart and ovaries. This would provide clinicians with more precise information for early diagnosis and staging.

Due to the patient’s arrhythmia and right atrial obstruction, the initial treatment plan of R-CHOP was reconsidered, as it could potentially increase cardiac stress. The treatment regimen was therefore adjusted to R-CEOD (27). This regimen works by inhibiting the proliferation of B-cell lymphoma through a combination of different drugs. After four cycles of treatment, the patient’s tumor burden was significantly reduced. Monitoring through ECG, echocardiography, and other diagnostic methods revealed no significant cardiac toxicity. Given the patient’s improvement, the treatment plan was switched back to R-CHOP to further consolidate the therapeutic effects. Based on the patient’s treatment response, future therapeutic strategies may incorporate targeted therapies, such as BTK inhibitors, and immunotherapy, such as PD-1 inhibitors. Studies have shown that BTK inhibitors are highly effective in treating B-cell-related malignancies and may reduce toxicity to sensitive organs, including the heart (28). PD-1 inhibitors, as immune checkpoint inhibitors, have demonstrated promising efficacy across various cancer types, potentially clearing tumor cells by activating the patient’s immune system, particularly in cases of multi-site metastasis (29, 30). Combining these targeted therapies and immunotherapies may further reduce tumor burden in metastatic sites such as the heart and ovaries, minimize treatment-related side effects, and improve patient survival. However, despite the theoretical promise of targeted therapies and immunotherapy, clinical application still requires more data from clinical trials. Future research should focus on evaluating the efficacy and safety of these treatment regimens in metastatic DLBCL patients to provide more personalized treatment options.

In this case, the presence of cardiac metastasis necessitates close monitoring of the patient’s cardiac function throughout the treatment. Given that DLBCL patients with cardiac involvement may face complications like heart failure during treatment, we suggest regular monitoring of cardiac function using techniques such as ECG and cardiac ultrasound before, during, and after treatment. Additionally, monitoring changes in cardiac function with specific cardiac biomarkers (like B-type natriuretic peptide and troponin) can facilitate the early detection of potential cardiac damage, enabling prompt intervention to avoid adverse events (31, 32).

This case offers valuable clinical insights into the rare multi-site metastasis of DLBCL, highlighting the need for more extensive multicenter studies to investigate the mechanisms of DLBCL metastasis. Future research could concentrate on the following aspects. First, utilizing high-throughput gene sequencing technology could help identify molecular markers associated with multi-site metastasis of DLBCL, such as specific chemokines and cell adhesion molecules (33–35). This could provide further insights into the specific factors that contribute to the formation of metastatic lesions in organs like the heart and ovaries. Second, investigating the mechanisms of interaction between DLBCL cells and the microenvironments of the heart and ovaries could help determine whether specific conditions in these microenvironments promote the growth of DLBCL cells (36). For instance, cells that metastasize to the heart may survive more readily in the environment of cardiac myocytes, while the local immune microenvironment in the ovaries might support the growth of tumor cells (37–39). Third, it is important to develop specific biomarkers and imaging screening techniques to facilitate the early detection of metastasis in rare sites of DLBCL (33, 40, 41). Building on this, a personalized follow-up and monitoring system for patients with DLBCL metastasis could offer more targeted treatment intervention strategies.

This case offers valuable clinical insights into the rare metastasis of DLBCL, emphasizing the importance of early recognition, careful monitoring, and personalized treatment for patients with multi-site metastasis. In the future, systematically collecting similar cases and establishing standards for the diagnosis, treatment, and follow-up of multi-site metastasis in DLBCL could provide reliable clinical evidence to enhance the prognosis of these complex metastatic patients.

## Conclusion

In a word, the findings of this case can provide insights into the individualized R-CEOD followed by R-CHOP regimen combined with multimodal imaging and histopathological evaluation for DLBCL patients with atypical cardiac and ovarian metastases. However, this study is limited by its single-case nature, and future multicenter prospective studies with larger cohorts are warranted to validate these clinical strategies and explore molecular mechanisms for optimizing targeted therapies such as BTK/PD-1 inhibitors.

## Data Availability

The original contributions presented in the study are included in the article/supplementary material. Further inquiries can be directed to the corresponding author.
